# M2 microglia-derived exosome-loaded electroconductive hydrogel for enhancing neurological recovery after spinal cord injury

**DOI:** 10.1186/s12951-023-02255-w

**Published:** 2024-01-03

**Authors:** Pengfei Guan, Lei Fan, Zhaobo Zhu, Qinfeng Yang, Xinchang Kang, Junji Li, Zuyu Zhang, Shencai Liu, Can Liu, Xuelian Wang, Jing Xu, Kun Wang, Yongjian Sun

**Affiliations:** 1 Department of Pediatric Orthopedic, Center for Orthopedic Surgery, the Third Affiliated Hospital of Southern Medical University, Guangzhou, 510515 China; 2 Department of Spine Surgery, Department of Orthopedics, Renji Hospital, School of Medicine, Shanghai Jiao Tong University, Shanghai, 200127 China; 3 Division of Orthopaedic Surgery, Department of Orthopaedics, Nanfang Hospital, Southern Medical University, Guangzhou, 510515 China; 4 Department of Orthopedic Surgery, Nanfang Hospital Baiyun Branch, Southern Medical University, Guangzhou, 510080 China; 5 Department of Biomedical Engineering, College of Life Science and Technology, Huazhong University of Science and Technology, Wuhan, 430074 China; 6 Department of Spine Surgery, Center for Orthopedic Surgery, the Third Affiliated Hospital of Southern Medical University, Guangzhou, 510515 China; 7 The Operating Room of the Third Affiliated Hospital of Southern Medical University, Guangzhou, 510515 China; 8 Department of Ophthalmology, Nanfang Hospital, Southern Medical University, Guangzhou, 510515 China

**Keywords:** Electroconductive hydrogels, M2 microglia, Exosomes, Immunomodulatory, Axon regeneration, Spinal cord injury

## Abstract

Electroconductive hydrogels offer a promising avenue for enhancing the repair efficacy of spinal cord injuries (SCI) by restoring disrupted electrical signals along the spinal cord’s conduction pathway. Nonetheless, the application of hydrogels composed of diverse electroconductive materials has demonstrated limited capacity to mitigate the post-SCI inflammatory response. Recent research has indicated that the transplantation of M2 microglia effectively fosters SCI recovery by attenuating the excessive inflammatory response. Exosomes (Exos), small vesicles discharged by cells carrying similar biological functions to their originating cells, present a compelling alternative to cellular transplantation. This investigation endeavors to exploit M2 microglia-derived exosomes (M2-Exos) successfully isolated and reversibly bonded to electroconductive hydrogels through hydrogen bonding for synergistic promotion of SCI repair to synergistically enhance SCI repair. In vitro experiments substantiated the significant capacity of M2-Exos-laden electroconductive hydrogels to stimulate the growth of neural stem cells and axons in the dorsal root ganglion and modulate microglial M2 polarization. Furthermore, M2-Exos demonstrated a remarkable ability to mitigate the initial inflammatory reaction within the injury site. When combined with the electroconductive hydrogel, M2-Exos worked synergistically to expedite neuronal and axonal regeneration, substantially enhancing the functional recovery of rats afflicted with SCI. These findings underscore the potential of M2-Exos as a valuable reparative factor, amplifying the efficacy of electroconductive hydrogels in their capacity to foster SCI rehabilitation.

**Supplementary Information:**

The online version contains supplementary material available at 10.1186/s12951-023-02255-w.

## Introduction

Spinal cord injuries (SCI) have profound and enduring consequences, often causing lasting motor and sensory impairments and inflicting enduring psychological, physical, and financial burdens on patients [1, 2]. The aftermath of SCI involves neuronal loss, and axon and myelin degeneration, coupled with the release of inflammatory signaling molecules like tumor necrosis factor (TNF-α) by activated microglia, which then triggers the activation of reactive astrocytes, culminating in the formation of a glial scar, impeding nerve and axon regeneration due to the unfavorable inflammatory environment and limited regenerative potential [3]. Effective SCI treatment necessitates the management of inflammation, fostering the recruitment of neural stem cells (NSCs), promoting neuronal regrowth, and guiding myelinated axon development [4].

Conventional clinical interventions for acute SCI include early fracture or dislocation reduction to restore spinal stability and high-dose intravenous methylprednisolone sodium succinate (MPSS) [5, 6]. However, this approach exhibits limited ability to stimulate axon and nerve regeneration, while excessive hormone doses can result in severe complications. The past few years have witnessed the advent of novel experimental strategies to foster axon growth in SCI repair, including cell transplantation and scaffold biomaterials [7–9]. Although cell transplantation has demonstrated huge promise in clinical studies, challenges such as unpredictable cell differentiation, low survival rates, and ethical concerns persist [10]. Given these issues, a compelling need arises for economical and cell-free biomaterial implants.

The application of scaffold biomaterial-based therapy, particularly electroconductive hydrogels, has emerged as a research hotspot for addressing SCI by bridging severed spinal cord ends [11, 12]. By generating an electric field akin to the native nervous system, these hydrogels facilitate the migration of endogenous NSCs into their matrix, steering their differentiation into neurons [13]. Additionally, mechanically pliable three-dimensional (3D) scaffolds facilitate neural tissue regeneration by creating an environment conducive to cell adhesion, growth, and differentiation [14]. However, the implantation of electroconductive hydrogels prompts immune responses, and the persistent presence of conductive components can exacerbate early inflammation post-acute SCI [14]. This sustained inflammatory reaction increases vacuolation and cyst formation in neural tissue, preventing restoration of neural function [15]. Furthermore, the excessive fibrous tissue encasing the conductive scaffold, induced by the inflammatory response, obstructs the integration of the hydrogel with the native spinal cord, hampering electrical signal conduction. Therefore, focusing exclusively on reestablishing electrical signal transmission through conductive scaffold bridging after spinal cord injury is insufficient to ensure comprehensive neurological recovery, as it neglects inflammation’s persistent and excessive nature.

Microglia, functioning as macrophages within the nervous system, ordinarily exist in a quiescent state (M0) and serve an immune monitoring role [16]. After SCI, microglia undergo structural and functional changes, eventually polarizing into M1 and M2 phenotypes. Activated M1 microglia secrete pro-inflammatory molecules like TNF-α and IL-1, triggering neuronal cell death, hindering axon regrowth, and causing demyelination [17]. Conversely, M2 microglia foster neuronal survival, axon extension, myelination, and synapse formation by releasing anti-inflammatory factors such as IL-4, IL-6, and IGF-1 [18]. M2 microglia transplantation has recently emerged as a promising avenue to enhance nerve regeneration and establish spinal cord homeostasis by modulating the inflammatory environment post-SCI [19]. Moreover, exosomes (Exos), measuring 30–150 nm in diameter and featuring a membrane-like structure, transport various miRNAs, lipids, and proteins, mirroring the functions of their parent cells [20]. Interestingly, Exos present a novel cell-free therapeutic approach that circumvents several limitations associated with cell transplantation, including immunogenicity and potential tumorigenicity [21, 22]. Consequently, introducing immunomodulatory exosomes derived from M2 microglia (M2-Exos) within an electroconductive hydrogel holds promise for attenuating adverse host immune responses and synergistically augmenting therapeutic outcomes to enhance functional recovery.

In this study, we developed electroconductive hydrogels composed of tannic acid (TA) and polypyrrole (PPy), incorporating exosomes with immunomodulatory properties. Initially, we engineered the TA/PPy (TP) hydrogel scaffold, possessing appropriate mechanical attributes and substantial electrical conductivity, through TA crosslinking and PPy doping. Robust intermolecular interactions, encompassing hydrogen bonds, electrostatic attractions, and hydrophobic interactions, facilitated the cross-linking and doping of the PPy network with TA. Subsequently, the reversible immobilization of M2-Exos was achieved within the TP hydrogel matrix, resulting in a TA/PPy/M2-Exos (TPME) hydrogel. Leveraging the abundance of polyphenolic groups within TA, M2-Exos could be tethered reversibly to the hydrogel matrix via hydrogen bonding. Furthermore, the non-covalent binding mechanism ensured the gradual and sustained release of exosomes during the initial phases of inflammation without compromising the structure and biological functionality of the exosomes. We comprehensively analyzed the micromorphology, physicochemical and electrical attributes, and the release and phagocytosis characteristics of M2-Exos within the TPME hydrogel. Furthermore, the impact of these hydrogels on the biocompatibility, proliferation, adhesion, and axonal regrowth of NSCs was assessed under in vitro conditions. To further elucidate the effects of TPME implantation on enhancing nerve regeneration and facilitating functional recovery following SCI, a rat model of complete spinal cord transection injury was established.

## Materials and methods

### Isolation and characterization of M2-Exos

For in vitro exosome studies, BV2 cells were selected due to their morphological and functional similarity to primary microglia, as demonstrated by Blasi et al. [23]. These BV2 cells, sourced from the American Type Culture Collection (ATCC) cell bank, were cultured in a high-glucose DMEM medium (Gibco), supplemented with 10% FBS (Gibco), with media replacement every two days. After PBS washing, BV2 cells were cultured for two days at 37 °C with 5% CO_2_ in fresh FBS supplemented with IL-4 (20 ng/mL) to generate M2 microglia. M2 microglia were further cultured in an exosome-free medium for another day before isolating M2-Exos through ultracentrifugation. Supernatants were subjected to sequential centrifugation steps (300 g, 3000 g, 10,000 g for 10 min each) to eliminate cell debris and live/dead cells. A final centrifugation step at 100,000 g for 90 min yielded M2-Exos, resuspended in phosphate-buffered saline (PBS) for preservation and analysis. M2-Exos morphology was assessed using transmission electron microscopy (TEM) (HT7700, HITACHI), and exosome size was determined via Nano Tracking Analysis (NTA) using the qNano system (Izon Science). A Western blot was conducted to identify surface marker expression, such as CD63 (ProteinTech) and TSG101 (Abcam), on M2-Exos. Red fluorescent dye PKH26 (Sigma-Aldrich) was used to label M2-Exos following the provided protocols.

### Synthesis of TPME hydrogel

Solution A was prepared by dissolving a specific amount of tannic acid in pure water to achieve a 0.6 wt% TA solution. Py was added to attain a final concentration of 0.8 mol/L. Solution B was prepared by combining 0.316 mg of ferric chloride hexahydrate (FeCl_3_·6H_2_O, 98%, Aladdin, Shanghai, China) with 0.6 ml of water. After briefly cooling solutions A and B in a 4 °C refrigerator, they were rapidly mixed to ensure a complete reaction. The remaining water-soluble reaction byproducts were soaked in ample pure water for three days to yield the TP hydrogel. Application of approximately 200 µg M2-Exos to the TP hydrogel’s surface resulted in the formation of TPME hydrogel.

### Characterization of the TPME hydrogel

#### Scanning electron microscopy (SEM)

The hydrogel sample was cryogenically treated with liquid nitrogen, and a cross-sectional segment was examined for micromorphology and structure using scanning electron microscopy (Quanta 200, FEI, USA).

#### Fourier transform infrared spectroscopy (FTIR)

The hydrogel’s composition was investigated using a Nicolet IS10 spectrometer (Thermo Scientific, USA). Scans were conducted by averaging 32 spectra per sample at a resolution of 4 cm^− 1^ across a range of 500–4000 cm^− 1^.

#### Rheological experiments

The hydrogel’s storage modulus (G’) and loss modulus (G”) were determined using a Physica MCR-301 rheometer (Anton Paar, Austria), scanning within a frequency range of 0.1–10 Hz. The measurements employed a PP-25 (25 mm) parallel plate detector.

#### Electrical properties

The TPME hydrogel’s electrical properties were assessed using a Zahner biennium electrochemical workstation (Germany). A silver/silver chloride (Ag/AgCl) electrode acted as the reference electrode, a pure platinum sheet functioned as the counter electrode, and a hydrogel deposited on an indium tin oxide (ITO) conductive glass substrate served as the working electrode. A 0.1 M phosphate-buffered saline (PBS) solution was used as the electrolyte. Cyclic voltammetry (CV) measurements were executed within a potential range of 0.8 to 1.0 V at a scan rate of 10 mV/s. AC impedance spectroscopy (EIS) analysis ranged from 0.01 Hz to 100 kHz. Electrical conductivity was assessed through two-probe current-voltage (I-V) measurements using a Keithley 2400 source meter (USA).

### In vitro studies

#### Neural stem cells culture

The hippocampus of E14 mouse embryos were dissociated and evenly triturated into single cells, which were subsequently cultured on a low-attachment platform. DMEM/F12 medium (Gibco) supplemented with epidermal growth factor (20 ng/ml), basic fibroblast growth factor (20 ng/ml), Glutamax, B27, and penicillin-streptomycin supported cell growth, with media renewal every three days.

#### Dorsal root ganglia (DRG) separation and cultures

Dorsal root ganglia (DRG) were extracted from 3-day-old newborn Sprague Dawley (SD) rats, following previous research procedures [13]. Briefly, pups were humanely euthanized, and the spinal column was dissected horizontally. Using stereomicroscopes, DRGs were meticulously isolated. Subsequently, these DRGs were cultivated on hydrogels in a serum-free medium consisting of neurobasal medium (Gibco), NGF (50 ng/mL), B27 neuronal supplement (1X, Gibco), penicillin/streptomycin (1X, Gibco), and Glutamax (1X, Gibco), all maintained at 37 °C. The culture medium was refreshed every other day.

#### Release profile and uptake of M2-Exos in TPME hydrogel

The release profile and uptake of M2-Exos in the TPME hydrogel were evaluated using a bicinchoninic acid (BCA) reagent test kit (Beyotime), as previously described in the literature [24]. In brief, pre-obtained TPME were incubated in PBS at 37 °C, with a total of 200 µg exosomes per sample. On days 1, 3, 7, and 14, the supernatant was collected to detect free exosomes using the BCA method. The exosome daily release profile and cumulative release profile were calculated by subtracting the number of exosomes in the supernatant from the total exosome amount.

Following a 24-hour co-culture, TPME and PC-12 cells were fixed with 4% paraformaldehyde and stained with Actin-Tracker Green (Beyotime) and Hoechst (Beyotime). Finally, confocal microscopy (Leica) was employed to visualize exosome phagocytosis.

#### In vitro biocompatibility of hydrogel

In vitro biocompatibility assessment encompassed cell viability, proliferation, and adhesion assays. Following a 24-hour co-culture of NSCs with each sample, NSC viability on the samples was determined using live/dead cell staining. Live cells were labeled with calcein-AM (Invitrogen), while dead cells were stained with propidium iodide (PI) (Invitrogen) to assess cell viability. The stained cells were observed using a confocal microscope (Leica).

NSC proliferation in each sample was evaluated using the cell counting kit 8 (CCK-8, Dojindo) on days 1, 3, and 7 of culture, as described in our previous study.

To assess NSC adherence, after 3 days of co-culture with each sample, cells were fixed in 4% paraformaldehyde. Cells were labeled with Actin-Tracker Green (Beyotime) and Hoechst (Beyotime), and their morphology was examined under a confocal microscope (Leica).

### Extraction of total RNA and real-time quantitative PCR

Total RNA was extracted from cells (Omega) following the kit instructions. The PrimeScript TM RT reagent Kit (Takara) was employed to perform reverse transcription of the complete RNA into cDNA. Real-time PCR was executed using SYBR staining and the QuantStudio 5 System (Thermo, USA). The relative expression level was calculated as 2^−ΔΔCt^ utilizing a standardized method. The experiment was conducted in triplicate, and Table S1 presents the primer sequences used.

### Immunofluorescence staining

Spinal cord tissues or NSCs were fixed using 4% paraformaldehyde for 30 min. Subsequently, they were permeabilized with 0.1% Triton X-100 (Biofroxx, Germany) for 10 min to disrupt the cell membrane, followed by blocking with 3% bovine serum albumin (BSA, Biofroxx, Germany) for 1 h at room temperature. After incubation with the primary and corresponding secondary antibodies, staining outcomes were visualized using confocal microscopy (Leica). Cell nuclei were counterstained with Hoechst (Beyotime). The list of primary antibodies utilized is provided in Table S2.

### Western blot

Spinal cord tissue or cells were evenly homogenized on ice in RIPA buffer (CWBIO, China), supplemented with protease and phosphatase inhibitors (Beyotime). The supernatants were collected following centrifugation at 12,000 rpm for 30 min at 4 °C. After determining the total protein content using the BCA kit, the supernatant was centrifuged at 12,000 rpm for 30 min at 4 °C. Proteins were separated on 8% SDS-PAGE gels and transferred onto PVDF membranes (Millipore). The membranes were blocked and incubated overnight at 4 °C with primary antibody, followed by two hours of incubation at 37 °C with secondary antibody. Three washes with 1x TBST (Biosharp) were conducted between each step. ImageJ software was used for data analysis. Table S2 lists the primary antibodies used.

### In vivo studies

#### Animals and surgical procedures

The study employed adult female Sprague Dawley (SD) rats weighing 200–250 g from the Experimental Animal Center of Southern Medical University. Complete cross-sections of spinal cord injury (SCI) were obtained according to established protocols [25]. Anesthesia was induced using a combination of 5 mg/kg xylazine and 70 mg/kg ketamine, and a 2–3 mm segment of T8-T10 spinal cord tissue was extracted. Following the spinal cord incision, the exosome-loaded electroconductive hydrogel was immediately transplanted into the spinal cord space. The rats were randomly divided into four groups (n = 10 per group): Sham, SCI, TP, or TPME. After surgery, catheterization was conducted until normal voiding function was regained. All experimental procedures adhered to the guidelines for animal care and use set by the National Institutes of Health and were approved by the Animal Care and Use Committee of Southern Medical University.

#### In vivo biocompatibility of hydrogel

At the end of the sixth week, the major organs (heart, liver, spleen, kidney, and lung) were extracted and fixed in formalin after sacrificing the rats in each group. Thin slices of these organ tissues were embedded in paraffin blocks and subjected to H&E staining, following the manufacturer’s instructions. Blood samples from each rat group were collected and centrifuged to obtain serum, which was then tested for alanine aminotransferase (ALT), aspartate aminotransferase (AST), and total protein levels. To assess hemolytic activity, rat whole blood and hydrogel were incubated at 37 degrees Celsius for 4 h. Both negative control (PBS) and positive control (Triton X-100) groups were included. Following incubation, the rat’s whole blood was centrifuged at 10,000 g for 5 min at 4 °C, and the optical density (OD) value of the supernatant was measured at 540 nm using an ultraviolet-visible spectrophotometer.

#### Behavioural tests of rats of SCI

The evaluation of hindlimb motor performance scores and footprint analysis were included in the behavioral assessment of rats with spinal cord injury. The Basso-Beattie-Bresnahan (BBB) motor function score was utilized to assess hindlimb motor function at various time points following SCI, including weeks 1 through 8. Blinded observers assessed and recorded the walking and limb activity scores of the hind limbs while the rat was placed on an open surface.

#### Magnetic resonance imaging (MRI) and diffusion tensor imaging (DTI)

Magnetic resonance imaging and diffusion tensor imaging were employed to evaluate spinal cord nerve tract regeneration in the sixth week, in accordance with a previous report. A 3.0 T MRI scanner (General Electric, USA) was utilized to examine the regeneration of spinal nerve fiber bundles. T2-weighted imaging (T2WI) and DTI were the primary indicators investigated. DTI data underwent processing and analysis to reconstruct and visualize tractography pathways.

#### Histological analysis

After six weeks, all rats were euthanized using an intraperitoneal injection of 3% sodium pentobarbital. The rat spinal cords, including the injured region, were fixed in 4% formalin buffer, embedded in paraffin, and sectioned into thin slices. Histological assessment was performed using hematoxylin and eosin (H&E) and Masson staining.

### Statistical analysis

GraphPad Prism 5 software was used for statistical analysis. The data were presented as mean ± standard deviation. Multiple measures were analyzed using one-way analysis of variance (ANOVA), followed by the Tukey post hoc test for multiple comparisons. Statistical significance was considered at p < 0.05.

## Results

### Characterization of M2-Exos

Previous research has demonstrated the efficacy of IL-4 in inducing microglia polarization toward the M2 phenotype [26, 27]. In the present study, immunofluorescence staining and Western blot analysis confirmed a substantial increase in Arg-1 expression in IL-4 treated BV2 cells, suggesting their transition from the M0 to the M2 phenotype (Fig. S1A, B). TEM revealed that the M2-Exos exhibited a cup-like morphology, while NTA revealed an approximate diameter of 120 nm (Fig. S1C, D). Western blot analysis (Fig. S1E) confirmed the presence of Alix, CD63, and TSG101, characteristic of exosomal traits, validating the successful synthesis of M2-Exos.

### Characteristics of TPME

**Fig. 1:**
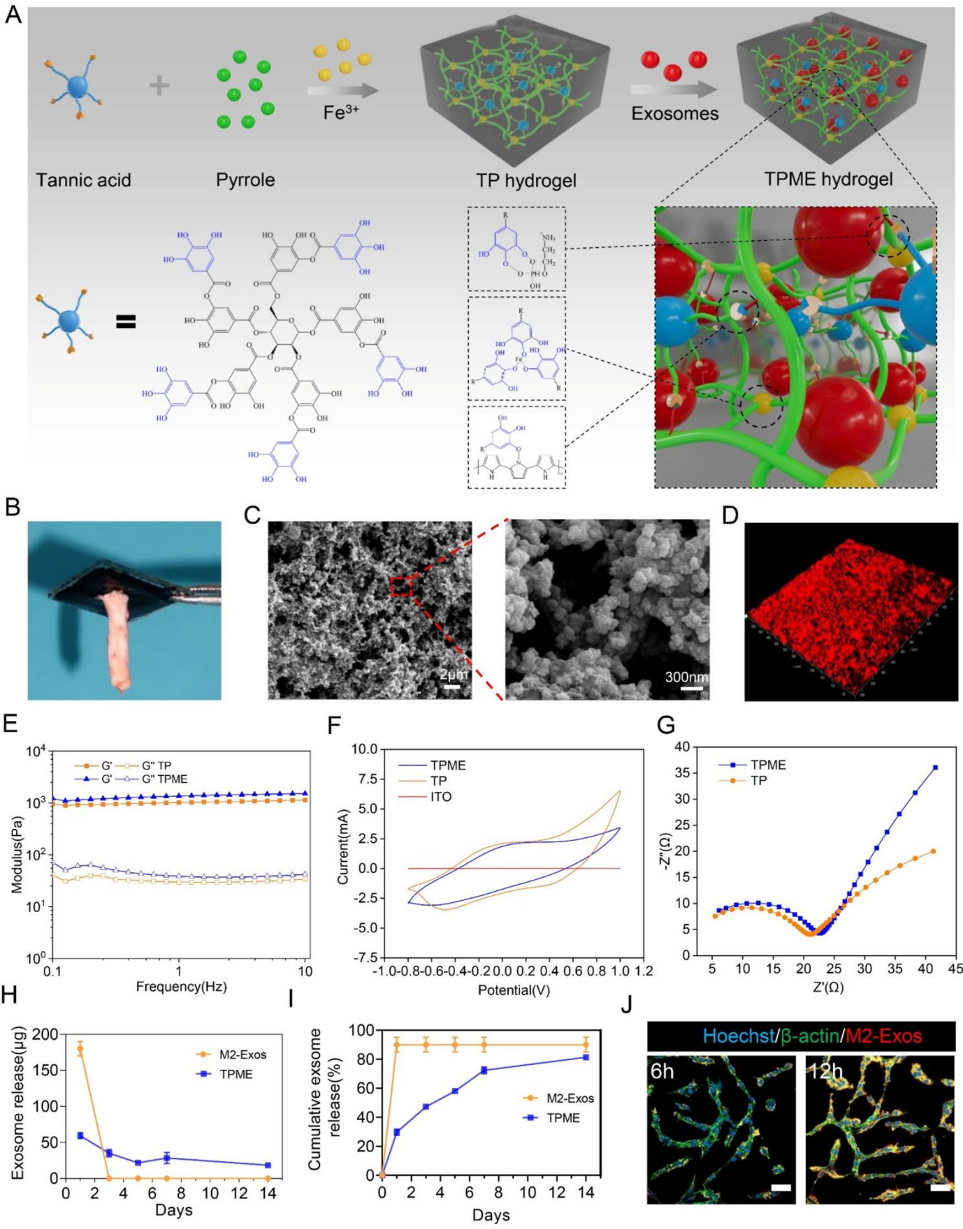
TP hydrogel properties when loaded with M2-Exos. (**A**) The two-step synthesis process of TPME hydrogels was illustrated. TP hydrogels were obtained by cross-linking with TA, Py, and Fe^3+^. TPME hydrogel synthesis occurred through the formation of hydrogen bonds between TA polyphenol groups and phosphate groups in the phospholipids of Exos. (**B**) Spinal cord tissue can adhere to the hydrogels in vitro. (**C**) SEM image of TPME hydrogel. (**D**) 3D image of TPME hydrogel with M2-Exos labeled with PKH26. (**E**) Rheological study of TP with and without Exos. (**F**) Cyclic voltammograms of TP and TPME hydrogels. (**G**) Nyquist curves of TP and TPME hydrogels. (**H**) Daily release curve of Exos with and without TP (n = 3). (**I**) The cumulative release profile of Exos with or without TP hydrogel over 14 days (n = 3). (**J**) Confocal microscopy image of the internalization of fluorescently labeled M2-Exos in PC12 cells. Scale bar = 50 μm. Statistical analysis was performed using ANOVA followed by Tukey’s test (*p < 0.05, **p < 0.01, and ***p < 0.001)

We employed a two-step synthesis to create TPME hydrogels (Fig. 1A). Initially, TP synthesis was accomplished by introducing an oxidation initiator (FeCl_3_) into a solution containing Py monomer and TA [28]. Subsequently, M2-Exos were immobilized within the TP hydrogel network to generate TPME hydrogel. The involvement of phenolic hydroxyl groups in TA was pivotal in this process. These groups not only engaged in intermolecular electrostatic interactions with protonated nitrogen groups on PPy, leading to cross-linking with PPy chains, but also demonstrated reversible binding with Exos through hydrogen bonding. The conductive properties of PPy originated from nitrogen atom protonation induced by TA [28]. The essential role of Fe^3+^ ions in gelation was apparent, as they facilitated Py polymerization and fostered the formation of ionic cross-linked networks with TA [29]. FT-IR spectra displayed a strong correlation between TP hydrogel and pristine PPy, confirming PPy as the principal component of the hydrogel (Fig. S2). The peak at 1174 cm^− 1^ in the TP spectrum was attributed to C-N + bond formation. Integrating Exos within the hydrogel did not modify the TP curve’s shape. Phenolic hydroxyl groups in TA facilitated TPME adherence to rat spinal cord tissue (Fig. 1B). SEM revealed that TPME exhibited a well-defined three-dimensional porous architecture composed of interconnected spherical nanoparticles, providing an extensive surface area for effective material exchange and cell adhesion (Fig. 1C). Moreover, this distinctive architecture facilitated cellular penetration and nerve ingrowth [28]. Additionally, 3D immunofluorescence (IF) imaging showcased uniform Exos distribution on the surface of TP hydrogels (Fig. 1D). Both hydrogels displayed markedly higher elastic moduli (G’) compared to loss moduli (G”), indicating their viscoelastic nature and stability. The mean elastic moduli exhibited no significant difference between TP (1017 ± 72 Pa) and TPME (1343 ± 127 Pa) (Fig. 1E). Cyclic voltammetry was applied to compare hydrogel electrochemical performance to bare ITO electrode. Both hydrogel types exhibited substantial current responses and amplified hysteresis loop regions, attributed to conductive PPy network formation within hydrogels (Fig. 1F). Moreover, electrochemical impedance spectroscopy unveiled a comparable high-frequency semicircle for both groups, indicating comparable charge-transfer resistances (Fig. 1G), which suggested that exosome incorporation did not compromise the excellent conductivity properties, and consistent conductivity with spinal cord tissue endorsed hydrogels’ suitability for electrical signal transmission. These findings underscore TPME’s alignment with spinal cord tissue’s mechanical properties (600–3000 Pa) and excellent electrical conductivity.

To assess exosome release from the hydrogel’s compatibility with the inflammatory response stage post-SCI, we recreated the in vivo conditions. The BCA method was employed to measure daily and cumulative exosome release profiles, revealing an initial concentration peak within the first week, followed by almost complete release over two weeks at a rate exceeding 80% (Fig. 1H, I). Following a 6-hour co-culture of TPME with PC12 cells, PKH26-labeled M2-Exos were identified within PC12 cells. Subsequently, their presence around the nucleus significantly increased at 12 h (Fig. 1J), indicating the efficient fusion of M2-Exos with PC12 cells.

### Evaluation of TPME biocompatibility

**Fig. 2:**
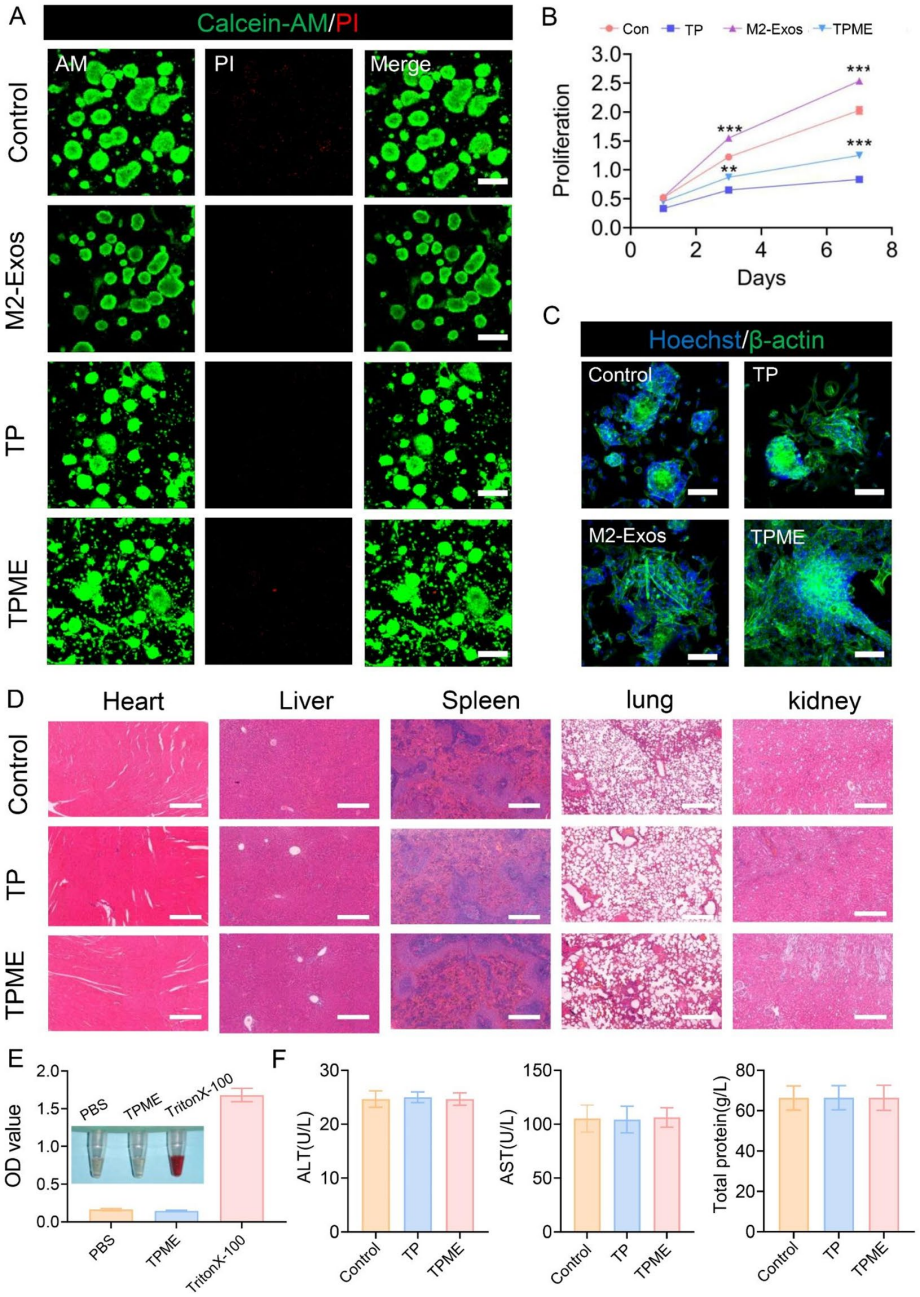
The biocompatibility of each group was evaluated. (**A**) Live/dead analysis was conducted after 1 day of cell culture. Green (AM-stained) and red (PI-stained) cells represented living and dead cells, respectively. Scale bar = 200 μm. (**B**) The CCK-8 test was performed in each group after 1, 3, and 7 days of cell culture (n = 5). (**C**) Cytoskeleton images of NSCs after 3 days of culture in each group. Scale bar = 100 μm. (**D**) HE stained images of the liver, kidney, heart, lung, and spleen of the sacrificed mice after 6 weeks of TP or TPME treatment. Scale bar = 400 μm. (**E**) Photograph of TPME-treated serum extracted from whole blood. Triton-100X and PBS were used as the positive and negative controls, respectively. This corresponds to the OD value of each group. (**F**) Serum levels of ALT, AST, and total protein in different groups. Statistical analysis was performed using ANOVA followed by Tukey’s test (*p < 0.05, **p < 0.01, and ***p < 0.001)

Staining of NSCs for viability exhibited a substantial proportion of live cells (green) and a negligible fraction of dead cells (red) across all sample sets, indicating that TP and Exos did not induce higher cytotoxicity (Fig. 2A). Additionally, the CCK-8 experiment demonstrated a consistent increase in NSC proliferation across all sample sets over time (Fig. 2B). Specifically, on days 3 and 7, the Exos group displayed significantly higher OD values compared to the control group, while the TPME group exhibited even greater NSC proliferation compared to the TP group (Fig. 2B). These results suggest that M2-Exos play a role in promoting NSC proliferation. Following three days of co-culture with NSCs, the cytoskeleton staining in the TPME group showed no changes compared to the control group. TP hydrogels functionalized with M2-Exos displayed significantly enhanced cell spreading compared to the TP group, indicating that the exosomes promoted cell adhesion and spreading (Fig. 2C).

Histological examination of organ sections (heart, liver, spleen, lung, and kidney) stained with H&E, as well as serum biochemical indices, showed no significant differences between each group and the control group at 8 weeks post-SCI in rats (Fig. 2D, F). Moreover, hemolysis experiments on the hydrogels revealed minimal hemolysis in the TPME group, similar to the PBS group. In contrast, the TritonX-100 group exhibited significant hemolysis due to damage to red blood cell membranes (Fig. 2E). The serum OD values in each group were consistent with the hemolysis test results. Based on the aforementioned findings, TPME hydrogels can be considered a safe therapeutic option for SCI.

### TPME promoted the M2 polarization of BV2 cells

To assess whether TPME hydrogels had immunomodulatory properties, GMPE hydrogels were co-cultured with BV2 cells for 3 days. After 3 days, the M1 phenotypic markers (TNF-α and iNOS) and M2 phenotypic markers (Arg-1 and IL-10) of each group were tested by qPCR and immunofluorescence. The mRNA expression levels of the anti-inflammatory cytokines Arg-1 and IL10 were significantly greater in the TPME group than in the TP group, according to the results of qPCR, whereas iNOS and TNF-α were significantly lower in the TPME group (Fig. S3A). The proportion of iNOS positive cells was lower in the TPME group compared to the TP group, but the proportion of Arg-1 positive cells was higher (Fig. S3B, C), which is consistent with our qPCR findings. These findings proved that M2-Exos encouraged M2 polarization in microglia.

### Axon outgrowth in NSCs and DRGs on TPME

**Fig. 3:**
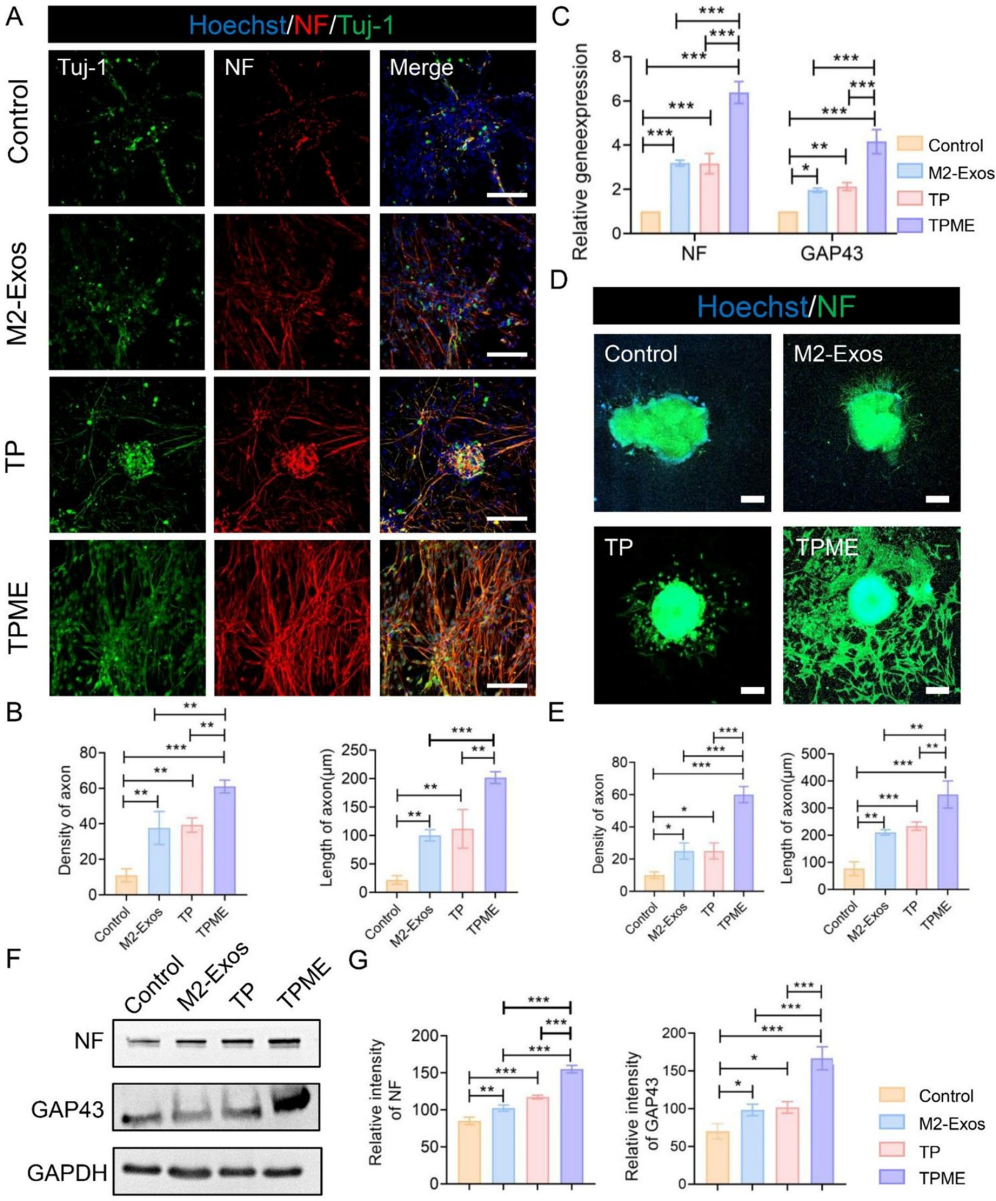
Axon outgrowth of NSCs and DRGs in vitro on various materials. (**A**) Confocal microscopy images showing the expression of neurofilament (NF, red) and β3-tubulin (Tuj-1, Green) in NSCs after 7 days of culture on different samples. Nuclei were stained with Hoechst (blue). Scale bar = 100 μm. (**B**) Quantification of axon density and axon length in NSCs grown on various materials (n = 3). (**C**) Gene expression profiles of NSCs cultured on different samples for 7 days (n = 3). (**D**) Immunofluorescence (IF) images of NF-positive axons in DRGs grown on different samples for 7 days. Scale bar = 200 μm. (**E**) Quantitative analysis of axon density and length in DRGs (n = 3). (**F**) Expression of GAP43 and NF proteins in NSCs cultured on different samples for 7 days. (**G**) Quantification of Western blot data (n = 3). Statistical analysis was performed using ANOVA followed by Tukey’s test (*p < 0.05, **p < 0.01, and ***p < 0.001)

To investigate the impact of hydrogels on regenerated axon growth in neural stem cells, we assessed the presence of neuronal-specific protein markers by IF staining: neurofilament (NF) and β3-tubulin (Tuj-1). After 7 days, substantial axon growth with NF-positive cells and Tuj-1-positive cells was observed in all NSC groups (Fig. 3A). Both the TP group and M2-Exos group exhibited higher axon density compared to the control group, with TPME hydrogels displaying the highest density (61 ± 3.61%). Additionally, the axon length of NSCs on TPME reached 201.67 ± 10.41 μm, surpassing that of the control group, TP group, and M2-Exos group (Fig. 3B). Quantitative analysis revealed that the relative expression levels of NF and growth-associated protein-43 (GAP43) genes in TPME were 6.38 ± 0.5-fold and 4.16 ± 0.54-fold higher than those in the control group, respectively. This enhancement primarily resulted from the axon-growth promoting effects of M2-Exos and TP (Fig. 3C). Western blot results corroborated the findings from immunofluorescence staining (IF) and quantitative PCR (qPCR), further confirming TPME hydrogel’s capability in promoting axon regeneration in NSCs (Fig. 3F, G). Moreover, considering the higher intrinsic potential of peripheral neurons compared to NSCs for axon regeneration studies, dorsal root ganglia (DRG) were isolated and analyzed. After 7 days of culture, NF staining of DRG revealed a significant increase in axon length and density in the TPME group compared to the control group, M2-Exos group, and TP group (Fig. 3D). Quantitative analysis indicated that both the TP group and M2-Exos group exhibited axon densities significantly higher than the control group but lower than the TPME group (Fig. 3E). Additionally, the axon length of TPME hydrogels measured 350 ± 50 μm, greater than the TP group (233 ± 15 μm), M2-Exos group (210 ± 10 μm), and control group (77 ± 25 μm) (Fig. 3E). Taken together, these findings demonstrate the significant stimulatory effect of both TP and M2-Exos present in TPME hydrogels on axon growth exerted.

### TPME improved motor function recovery after SCI in rats

**Fig. 4:**
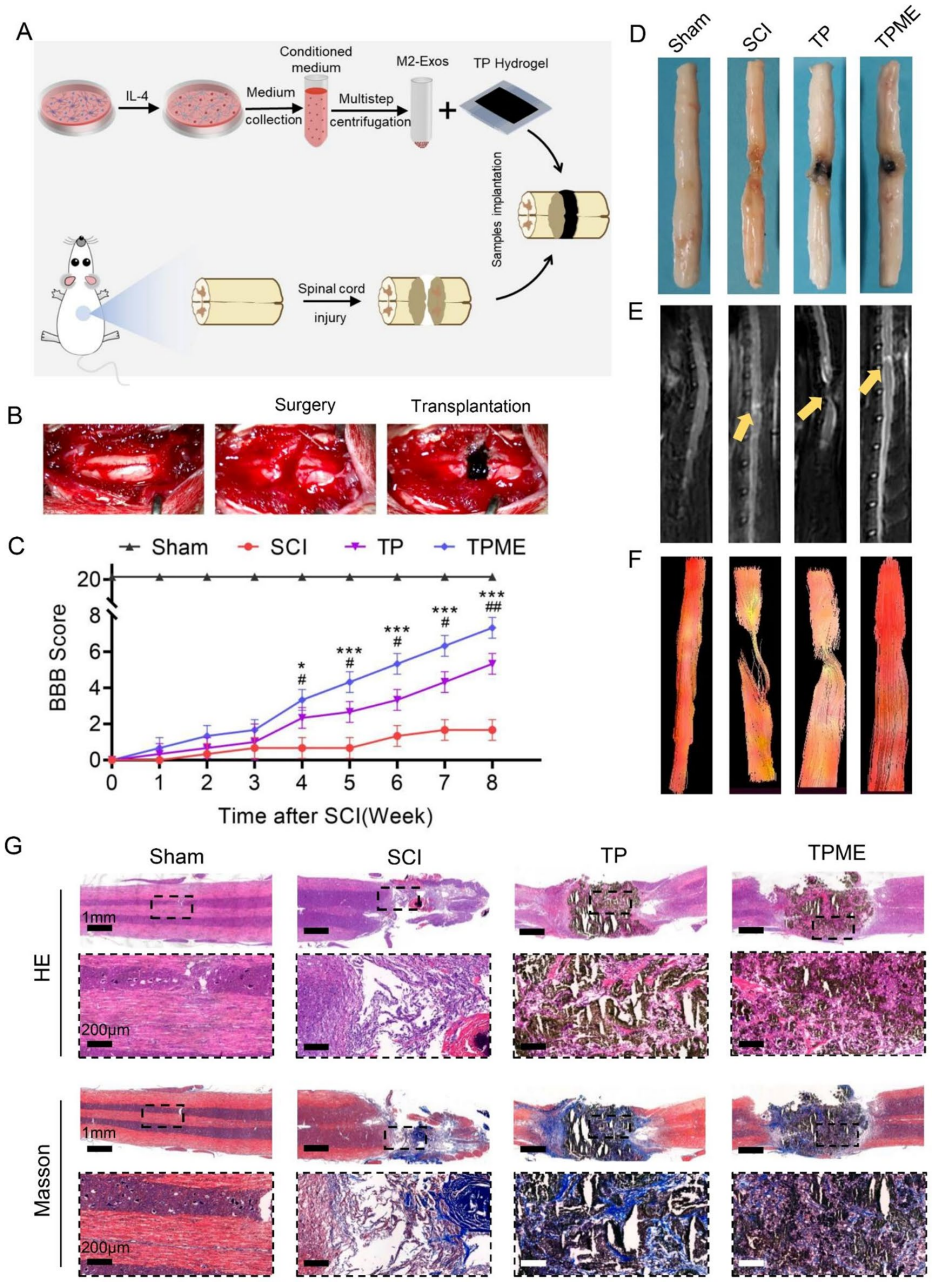
TPME implant enhances functional recovery. (**A**–**B**) Diagram illustrating the process of SCI and hydrogel implantation. (**C**) Locomotor recovery in rats was evaluated using the BBB score in an open field (n = 3). (**D**) Representative spinal cord images from all groups after SCI repair. (**E**–**F**) MRI and DTI images from all groups showed healing of spinal cord damage. Yellow arrows indicate the location of the SCI. (**G**) Representative images of hematoxylin-eosin staining and Masson’s trichrome staining illustrate the spinal cord morphology in different groups. Statistical analysis was performed using ANOVA followed by Tukey’s test (*p < 0.05, **p < 0.01, and ***p < 0.001)

In the present study, a complete rat spinal cord transection model was employed to assess the effect of TPME implantation after SCI. Indeed, complete SCI accurately represents axonal regeneration, as opposed to potential axonal sprouting observed in certain traumatic SCI models [30]. The surgical procedure and schematic diagram are depicted in Fig. 4A and B. Due to its robust tissue adhesion, the hydrogel patch functioned as a highly conductive bridge, closely connecting with the spinal cord tissue (Fig. 4B). Motor function recovery in rats after SCI was evaluated using BBB scores (Fig. 4C). Following surgery, all rats exhibited complete hindlimb paralysis (BBB score = 0). Until the third week post-injury, no significant differences were observed between the TPME and SCI groups. Starting from the fourth week post-surgery, the TPME group demonstrated higher scores than the TP group, indicating the beneficial impact of M2-Exos on functional recovery. Over eight weeks, certain TPME-treated rats achieved a BBB score of 8 points, whereas the SCI group’s BBB score did not exceed 2 points. Importantly, the re-establishment of nerve connections at the injury site facilitates the restoration of disrupted electrical signals, a pivotal factor in rat motor function recovery. These findings suggest that M2-Exos could enhance TP hydrogels’ efficacy in promoting motor function recovery after SCI. Gross and MRI images of the spinal cord at 8 weeks post-injury are shown in Fig. 4D and E. Figure 4D illustrates that the untreated group exhibited fewer neural connections at the injury site, potentially contributing to slower motor function recovery in the SCI group. In contrast, the TPME group displayed superior nerve regeneration within the injury area and enhanced compatibility between the hydrogel and spinal cord tissue compared to the TP group, likely due to an improved inflammatory microenvironment. Subsequent MRI images, especially diffusion tensor imaging, indicated reduced nerve tissue presence at the lesion site in the SCI group, while the TPME group showed significant nerve fiber infiltration and growth (Fig. 4F). H&E and Masson’s staining demonstrated that the TPME hydrogel facilitated nerve fiber ingrowth, while nerve fibers in the SCI group appeared disorganized and interrupted (Fig. 4G). Additionally, TPME exhibited reduced fibrous tissue surrounding it, further promoting hydrogel integration with spinal cord tissue and enhancing axonal growth and electrical signaling. These outcomes indicated that implanting TPME hydrogel at the severed end of the spinal cord could enhance nerve tissue regeneration, reinstate electrical signal conduction, and improve motor recovery capacity.

### TPME promoted neurogenesis at the injury site

**Fig. 5:**
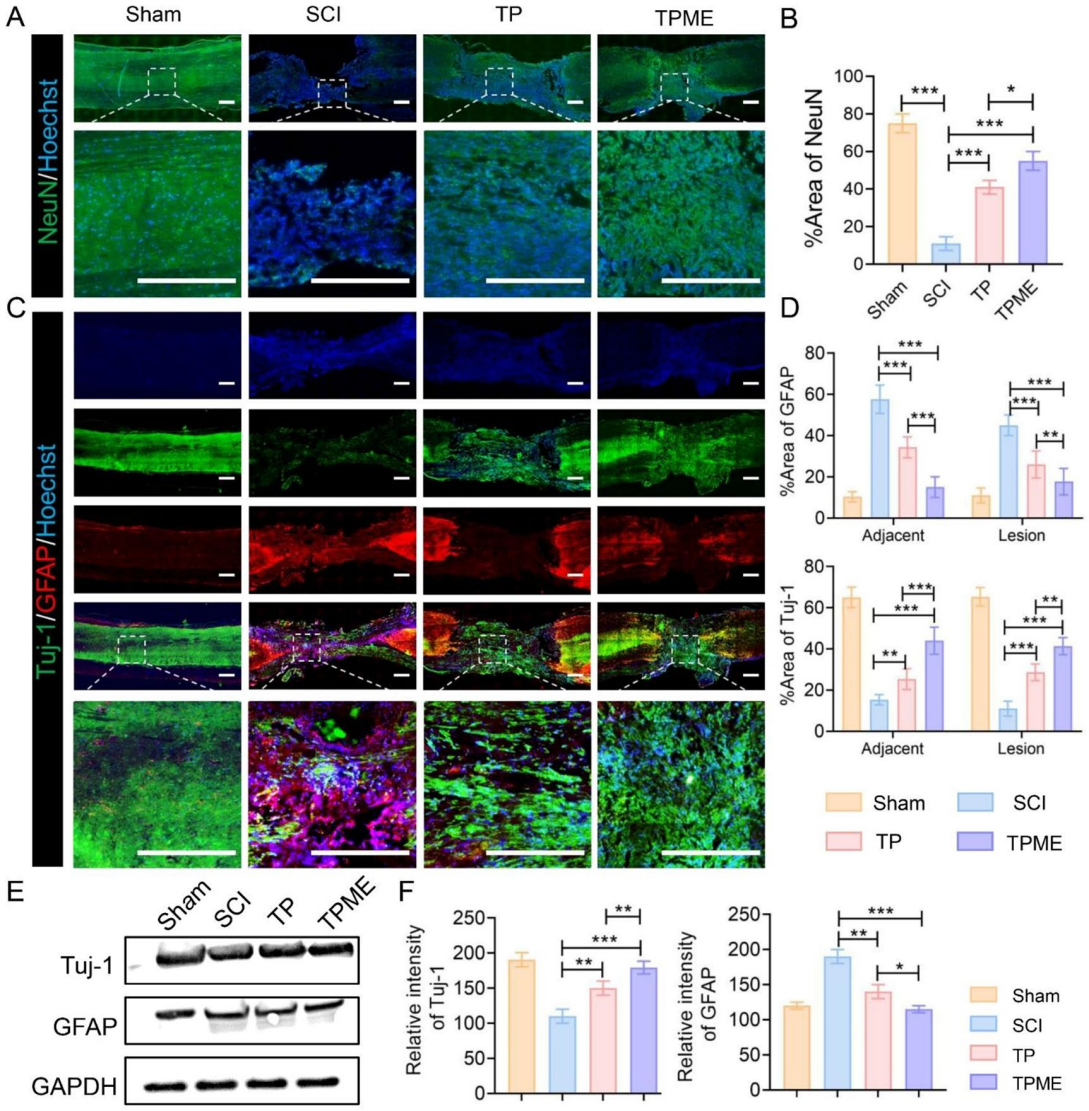
Endogenous neurogenesis is enhanced by TPME implantation. (**A**) Representative photograph of NeuN (green) staining 8 weeks after SCI in different experimental groups. Scale bar = 500 μm. (**B**) Quantitative analysis of the area occupied by NeuN-positive cells (n = 3). (**C**) Immunofluorescence images from the various groups at 8 weeks. Neuronal markers (Tuj-1, green) and glial fibrillary acidic protein (GFAP, red) were used to stain the spinal cords. Scale bar = 500 μm. (**D**) Quantitative study of Tuj-1 and GFAP immunofluorescence intensity in spinal cord tissue (n = 3). (**E**) Tuj-1 and GFAP protein expression. (**F**) Quantitative analysis of Tuj-1 and GFAP in Western blotting studies (n = 3). Statistical analysis was performed using ANOVA followed by Tukey’s test (*p < 0.05, **p < 0.01, and ***p < 0.001)

To explore the mechanisms underlying rat motor function recovery after SCI at 8 weeks, immunobiological assays were conducted on spinal cord specimens. NeuN, a neuron-specific nuclear protein, was employed to assess neuron survival and recovery post-spinal cord injury. The TPME group displayed a higher count of NeuN-positive cells at the injury site than the SCI and TP groups (Fig. 5A, B). Subsequently, Tuj-1 (β3-tubulin) and GFAP (glial fibrillary acidic protein) staining were employed in the injured area and its vicinity. The untreated group exhibited fewer nerve connections and a substantial cavitated region, whereas treatment groups exhibited reduced cavity size and increased nerve connections (Fig. 5C). However, the defect area exhibited minimal variation among all groups, suggesting that treatment facilitated the migration of endogenous neural stem cells. In contrast to the SCI group, the TP and TPME groups exhibited numerous Tuj1-positive cells at the lesion site and adjacent regions, indicating a higher incidence of new neuron formation. Conversely, the SCI group demonstrated significant accumulation of reactive astrocytes at the lesion site, which hindered nerve fiber penetration and reconstruction, a phenomenon not observed in the TP and TPME groups. Quantitative analysis demonstrated significantly higher levels of Tuj-1-positive neurons at the injury site in the TP group (28.67 ± 4.04%) and TPME group (41.33 ± 4.16%) in comparison to the SCI group (11 ± 3.61%) (Fig. 5D). Conversely, the proportion of GFAP-positive cells displayed the opposite trend, suggesting that hydrogel treatment attenuated astrocyte accumulation at the injury site (Fig. 5D). Western blot assays further validated the immunofluorescence results (Fig. 5E, F). These findings suggest that TPME hydrogel promotes endogenous neurogenesis and reduces glial scar formation at the spinal cord injury site.

### TPME promoted axonal regeneration and remyelination at the site of injury

**Fig. 6:**
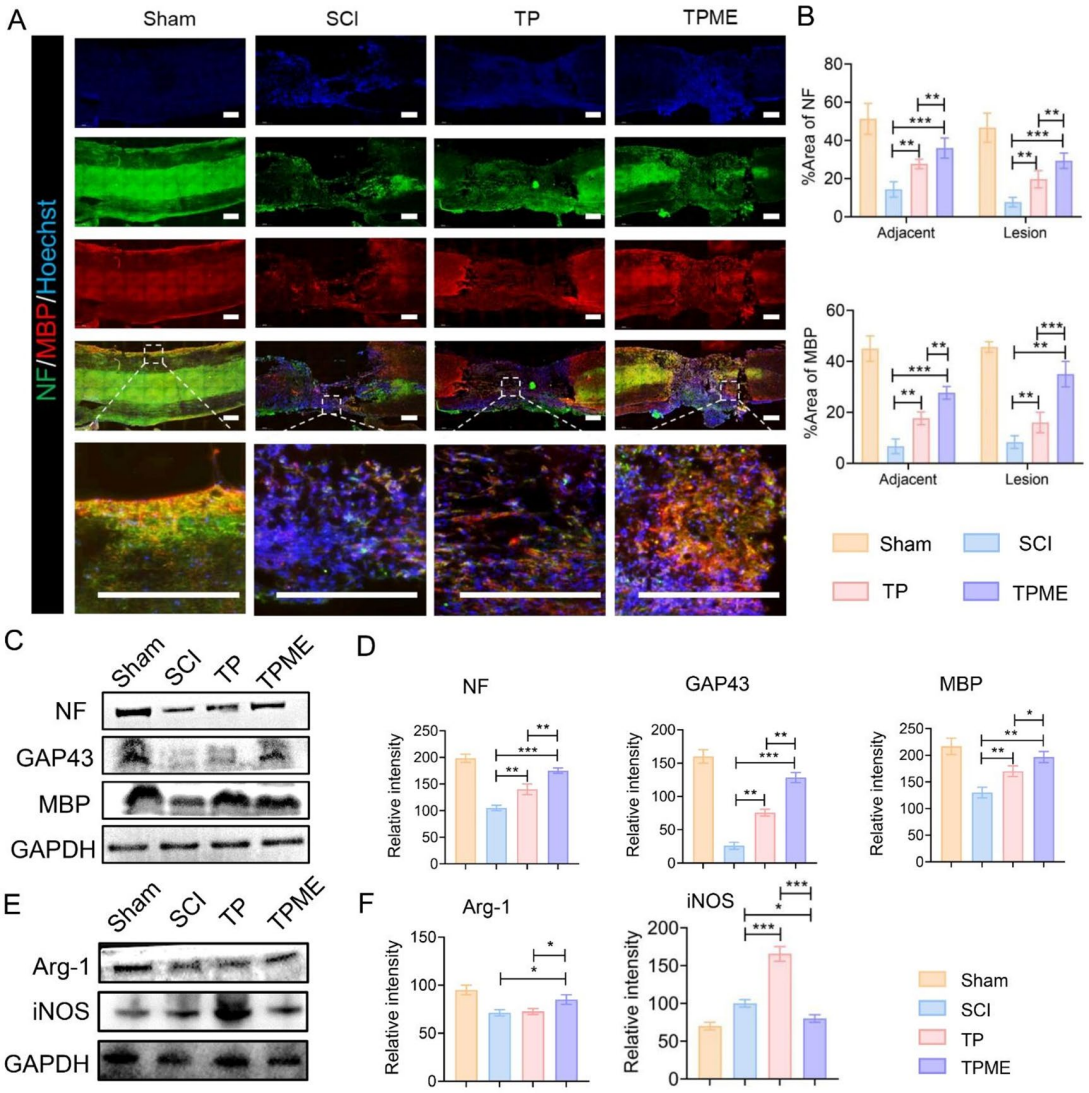
TPME implant enhances axon regeneration and remyelination. (**A**) Immunofluorescence images of separate groups at 8 weeks. Neurofilament (NF, green) and myelin basic protein (MBP) antibodies were utilized to stain the spinal cords (MBP, red). Scale bar = 500 μm. (**B**) Quantitative analysis of NF and MBP immunofluorescence intensity in spinal cord tissue (n = 3). (**C**) Protein expression of NF, GAP43, and MBP. (**D**) Quantitative analysis of NF, MBP, and GAP43 in Western blotting assays (n = 3). (**E**) Protein expression of Arg-1 and iNOS. (**F**) Quantitative analysis of Arg-1 and iNOS in Western blotting assays (n = 3). Statistical analysis was performed using ANOVA followed by Tukey’s test (*p < 0.05, **p < 0.01, and ***p < 0.001)

It is now understood that the formation of mature nerve fibers and myelin is crucial for functional recovery post-spinal cord injury. NF and myelin basic protein (MBP) labeling were utilized to identify regenerated axons and myelin at the damage site. Figure 6A depicts that the SCI group’s lesion and nearby regions exhibited only fragmented, sheet-like extension of axon fibers, whereas both TP and TPME groups displayed continuous and extensive infiltration of NF-positive fibers. Importantly, the TPME group exhibited more NF-positive fibers than the TP group, indicating M2-Exos’ beneficial effect on nerve fiber survival in the conductive hydrogel. Correspondingly, the TPME group displayed the highest count of MBP-positive cells, suggesting that the electroconductive hydrogel’s electrical milieu and anti-inflammatory environment were conducive to nerve axon remyelination. For quantitative analysis, the area ratios of NF and MBP at the lesion sites were 29.33 ± 4.04% and 35 ± 5%, 19.67 ± 4.51% and 16 ± 4%, 7.67 ± 2.52% and 8.33 ± 2.52% in the TPME, TP, and SCI groups, respectively (Fig. 6B). Additional Western blot tests revealed that compared to the SCI group, expression of NF, MBP, and GAP43 was highest in the TPME group, followed by the TP group (Fig. 6C, D). Moreover, macrophage/microglia polarization was assessed through markers iNOS and Arg-1 in the lesion area. As depicted in Fig. 6E and F, Arg-1 marker expression significantly increased in M2 microglia after M2-Exos addition compared to TP treatment alone. In contrast, iNOS expression suggested that TP grafting exacerbated local inflammation, whereas M1 microglia marker expression decreased after M2-Exos loading. These results suggest that M2-Exos increased the M2/M1 ratio in the lesion area, promoting macrophage/microglia conversion to the M2 phenotype. Conversely, TP grafting failed to alleviate inflammation and exacerbated it post-injury.

**Fig. 7:**
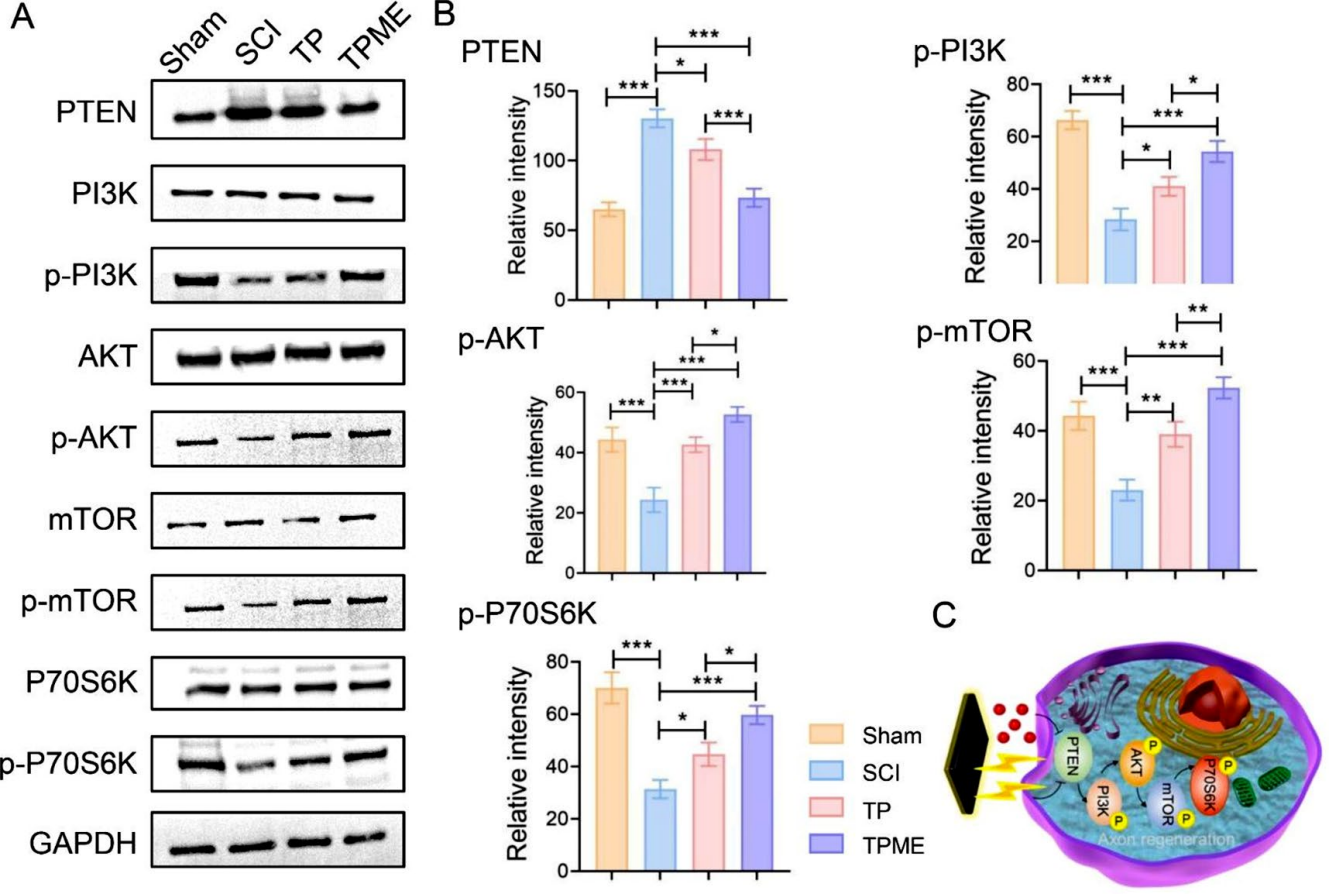
Mechanism of TPME hydrogels in promoting axon regeneration after SCI. (**A**) The expression of PTEN/PI3K/AKT/mTOR pathway proteins was detected using Western blotting. (**B**) Western blotting assays were used to quantitatively analyze PTEN, p-PI3K, p-AKT, p-mTOR, and p-P70S6K (n = 3). (**C**) A schematic illustration demonstrates that the mechanism by which TPME hydrogels promote axon regeneration is through the activation of the PTEN/PI3K/AKT/mTOR pathway. Statistical analysis was performed using ANOVA followed by Tukey’s test (*p < 0.05, **p < 0.01, and ***p < 0.001)

The mammalian target of rapamycin (mTOR) signaling pathway is typically inhibited by negative regulators such as PTEN (phosphatase and tension homolog gene on chromosome 10), potentially contributing to the failure of central nerve regeneration [14]. Therefore, relative protein expression of the PTEN/phosphatidylinositol 3-kinase (PI3K)/protein kinase B (AKT)/mTOR pathway was assessed through Western blot (Fig. 7). PTEN levels were notably lower in the TP and TPME groups than in the SCI group. While phosphorylated PI3K (p-PI3K), p-AKT, p-mTOR, and p-P70S6K expression significantly increased in the TP and TPME groups, the overall levels of these proteins remained consistent under different growth conditions. Moreover, p-PI3K, p-AKT, p-mTOR, and p-P70S6K levels were higher in the TPME group than in the TP group, while PTEN levels were lower in the TP group. These findings suggest that these axonal regeneration processes are linked to PTEN/PI3K/AKT/mTOR pathway activation. Collectively, these findings indicate that conductive hydrogels work synergistically with M2 microglia-derived exosomes to promote axon regeneration and remyelination post-spinal cord injury.

## Discussion

Neuroinflammation, glial scar formation, and challenges in axonal regeneration are major factors that hinder the regenerative potential following spinal cord injury [31, 32]. Biomaterial-based scaffold treatments, particularly hydrogels capable of conducting electrical signals, have emerged as promising strategies to promote axonal regrowth and mitigate scar formation [33, 34]. Our developed TP hydrogel exhibited high electrical conductivity (10^− 6^-10^− 5^ S/cm), outperforming common conductive hybrid hydrogels (10^− 6^-10^− 5^ S/cm) [28]. Crucially, this hydrogel’s conductivity was consistent with that of spinal cord tissue (10^− 2^-10^− 1^ S/cm), rendering it suitable as a scaffold for reconnecting severed spinal cord ends [35]. Furthermore, our hydrogels demonstrated mechanical properties, including a Young’s modulus of 1343 ± 127 Pa, comparable to those of spinal cord tissues (100–3000 Pa), positioning them as promising candidates for nerve damage recovery [28, 29]. It is widely acknowledged that material mechanical properties can influence neural stem cell fate, with soft materials (0.1-1 kPa) promoting neuron differentiation and slightly more rigid materials (7–10 kPa) favoring glial cell differentiation [23]. The storage modulus of the TP hydrogel in our study (1100 ± 15 Pa) corresponds to neural tissue mechanics (600–3000 Pa), establishing its suitability for soft nerve tissue engineering. Importantly, our previous work demonstrated that this conductive polymer hydrogel exhibited in vivo degradation, enabling nerve cell migration into the hydrogel interior [28].

Earlier studies have revealed that hydrogel electrical conductivity can directly activate voltage-gated Ca^2+^ channels, elevating intracellular Ca^2+^ levels [14]. Subsequently, the PTEN protein is negatively regulated through calcium/calmodulin-dependent protein kinase II (CaMKII) activation, boosting mTOR activity and fostering axonal regrowth in corticospinal neurons [36, 37]. Electroactive conductive hydrogels have also been shown to stimulate axon extension and myelin formation by activating PI3K/AKT and MEK/ERK pathways and promoting endogenous NSC migration to injury sites [13, 14, 29]. The present study demonstrated that, in addition to TP hydrogels activating the PTEN/PI3K/AKT/mTOR pathway, M2-Exos could inhibit PTEN expression, synergistically promoting axonal regeneration with TP hydrogels. Consistently, other studies confirmed that M2-Exos induce the shift of M1 macrophages into the M2 phenotype by stimulating PI3K/AKT, consistent with our study findings [38].

Following SCI, activation of A1-reactive astrocytes and M1 microglia leads to neuronal apoptosis, delayed axonal regrowth, and demyelination [18]. Severe inflammatory responses also lead to a material-spinal cord tissue mismatch due to extensive fibrosis around the material, impeding bioelectrical signal transmission. Consequently, persistent inflammation accelerates nerve damage and diminishes the therapeutic effect of conductive biomaterials. In recent years, a form of direct immunotherapy has emerged, where M2 microglia, generated from IL-4, IL-10, or TGF-β1, are transplanted into injured spinal cords [39]. This strategy promotes a shift of local microglia/macrophages towards the M2 phenotype, thereby improving myelin repair and neuronal survival and facilitating the functional restoration of neurons [19]. However, cell transplantation is limited by availability, tumor growth risks, disease transmission concerns, and potential allograft-induced immune reactions. Recent studies have highlighted Exos as paracrine-secreted intercellular communication mediators. It has been established that M2-Exos carry numerous anti-inflammatory and repair factors from parent cells [38]. Wang et al. utilized an injectable hydrogel for sustained M2-Exos release, promoting spinal cord regeneration by inducing local M2 microglia polarization and inhibiting neuronal death via the Bcl-2 pathway [40]. Furthermore, M2-Exos can stimulate Wnt/β-catenin signaling, fostering vascular regeneration post-SCI and ultimately promoting spinal cord nerve regeneration [41]. Thus, to address deleterious biological responses after secondary injury, we incorporated M2-Exos into hydrogels, conferring anti-inflammatory and neuroprotective properties and enhancing functional recovery post-spinal cord injury. Utilizing Exos for local release could further enhance hydrogel efficacy, potentially extending their applications across various medical fields.

Our findings provide compelling evidence that the incorporation of M2-Exos into hydrogels does not compromise the original electrical activity of the hydrogel or alter its mechanical properties, which are akin to those of spinal cord tissue. Importantly, M2-Exos, a bioactive agent, enhances the compatibility of TP hydrogels with NSCs. Furthermore, most Exos within the hydrogel are released within the initial 7 days, aligning with the pathological timeline following SCI. Unlike conventional hydrogels that often exhibit burst release of encapsulated substances, our system’s stable encapsulation of M2-Exos prevents such rapid release, attributed to the protective environment maintained at 37 °C. CCK-8 studies revealing the added value of NSCs underscore the supportive role of M2-Exos. This enhancement could be attributed to miRNAs delivered by Exos, potentially stimulating AKT and ERK signaling pathways [24]. Notably, M2-Exos exert effective control over the highly inflammatory milieu and diminish fibrosis surrounding the hydrogel post-spinal cord injury, markedly expediting spinal cord function recovery. Compared to hydrogel grafts in isolation, hydrogels laden with M2-Exos exhibit heightened neuronal survival at the injury site and attenuated development of glial scarring. Additionally, the TPME group exhibited a noteworthy increase in myelinated nerve fibers, a consequence closely associated with the dampening of inflammation at the injury site. Ultimately, obtaining electrophysiological data from neurons on acute spinal cord sections through membrane attachment or whole-cell recording using patch clamp techniques could offer more direct insights into functional recovery. However, this specific information is presently absent in our study. We will explore the possibility of incorporating this aspect into future investigations or collaborating with experts in the field if deemed feasible. In conclusion, M2-Exos, harnessing their anti-inflammatory and pro-axonal regenerative attributes, emerge as a promising option for supplementary conductive hydrogels in spinal cord injury treatment.

## Conclusion

Herein, we innovatively combined M2-Exos, which have well-established anti-inflammatory effects, with an electroconductive hydrogel to address spinal cord injury. Through reversible non-covalent binding, M2-Exos were immobilized within TPME hydrogels, enabling sustained release over approximately two weeks while maintaining their efficacy. Compared to the injury-only group, the implantation of TP hydrogel promoted the regeneration of neurons and myelinated axons but failed to alleviate and, in some cases, exacerbated the inflammatory response at the injury site during the early stages post-SCI. The introduction of M2-Exos, however, led to a significant reduction in the post-injury inflammatory response, fostering neuronal survival and mitigating the formation of glial scars. Furthermore, M2-Exos played a role in dampening the fibrotic response induced by hydrogel implantation, thus facilitating the seamless integration of the hydrogel with spinal cord tissue. Notably, the collaborative effect of M2-Exos within the electroconductive hydrogel synergistically promoted axon growth, primarily through the initiation of the PTEN/PI3K/AKT/mTOR signaling pathway. Consequently, our innovative approach involving electroconductive hydrogels combined with M2-Exos, equipped with immunomodulatory and pro-axonal regenerative attributes, holds significant promise as a therapeutic strategy to enhance functional recovery following spinal cord injury.

### Electronic supplementary material

Below is the link to the electronic supplementary material.

**Supplementary Material 1:** IL-4 modulated BV2 cell polarization and characteristic of extracellular vesicles derived M2 type BV2 cells

## Data Availability

All data generated or analysed during this study are included in this published article.
