# Supplementary material for: M2 microglia-derived exosome-loaded electroconductive hydrogel for enhancing neurological recovery after spinal cord injury
Source: J Nanobiotechnology. 2024 Jan 3;22:8. doi: 10.1186/s12951-023-02255-w (PMC10763283; doi:10.1186/s12951-023-02255-w)
Supplement: Supplementary file 1 — Supplementary Material 1: IL-4 modulated BV2 cell polarization and characteristic of extracellular vesicles derived M2 type BV2 cells [file 12951_2023_2255_MOESM1_ESM.docx]

M2 microglia-derived exosome-loaded electroconductive hydrogel for enhancing neurological recovery after spinal cord injury

*Pengfei Guan^1, *^, Lei Fan^3, *^, Zhaobo Zhu^4, *^, Qinfeng Yang^3, *^, Xinchang Kang^5^, Junji Li^1^, Zuyu Zhang^1^, Shencai Liu^3^, Can Liu^6^, Xuelian Wang^7^, Jing Xu^8, #^,* *Kun Wang^2, #^, Yongjian Sun^1, #^*

^1^Department of Pediatric Orthopedic, Center for Orthopedic Surgery, the Third Affiliated Hospital of Southern Medical University, Guangzhou, 510515, China

^2^Department of Spine Surgery, Department of Orthopedics, Renji Hospital, School of Medicine, Shanghai Jiao Tong University, Shanghai, 200127, China

^3^Division of Orthopaedic Surgery, Department of Orthopaedics, Nanfang Hospital, Southern Medical University, Guangzhou, 510515, China

^4^Department of Orthopedic Surgery, Nanfang Hospital Baiyun Branch, Southern Medical University, Guangzhou, 510080, China

^5^Department of Biomedical Engineering, College of Life Science and Technology, Huazhong University of Science and Technology, Wuhan, 430074, China

^6^Department of Spine Surgery, Center for Orthopedic Surgery, the Third Affiliated Hospital of Southern Medical University, Guangzhou, 510515, China

^7^The operating room of the Third Affiliated Hospital of Southern Medical University, Guangzhou, 510515, China

^8^Department of Ophthalmology, Nanfang Hospital, Southern Medical University, Guangzhou 510515, China

* The first three authors contributed equally to this work.

# Corresponding author. Email Address: [nysysyj@163.com](mailto:nysysyj@163.com) (Yongjian Sun); xjophthal@smu.edu.cn (Jing Xu); wk52693158@126.com (Kun Wang).


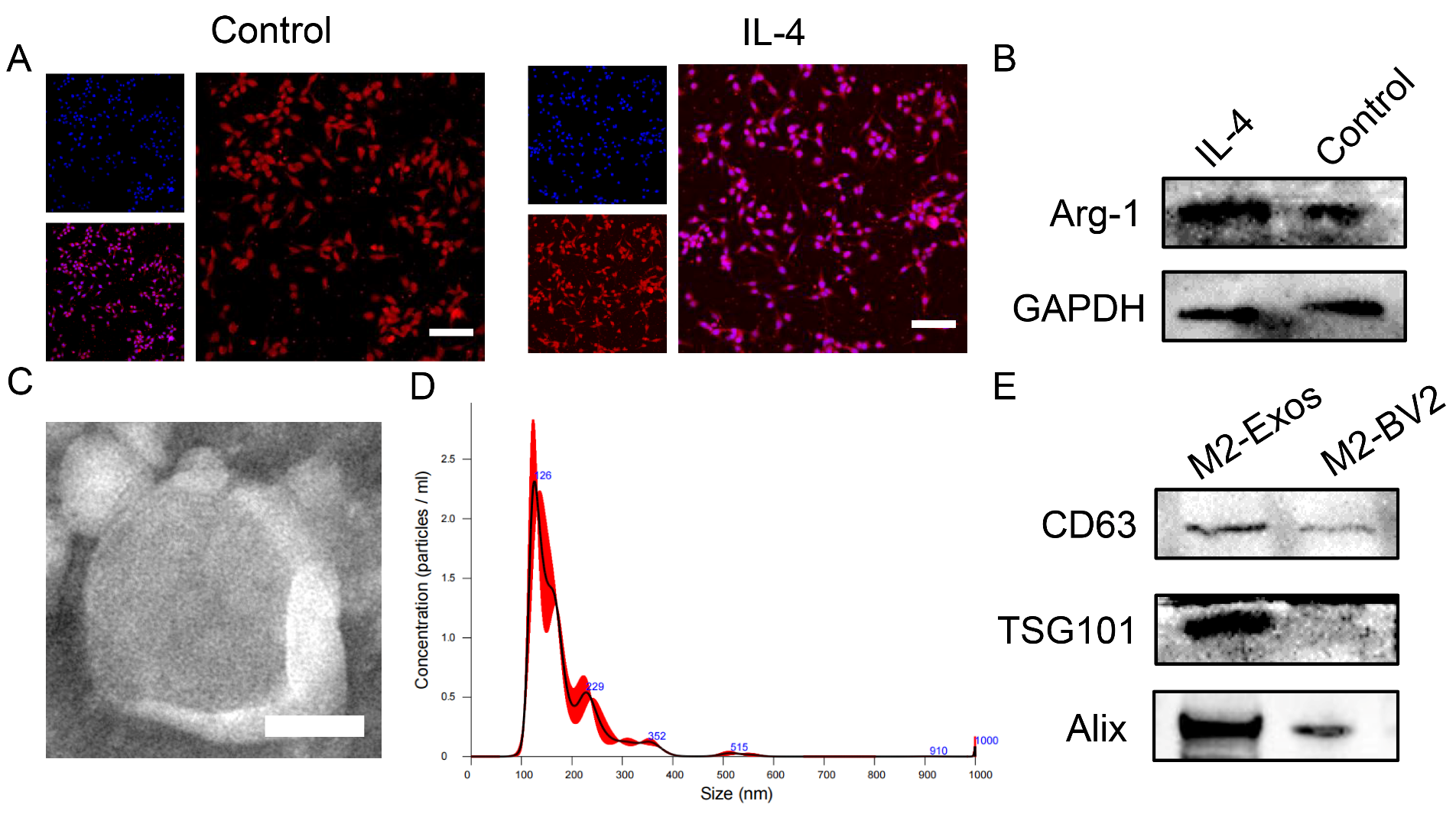


Fig. S1 IL-4 modulated BV2 cell polarization and characteristic of extracellular vesicles derived M2 type BV2 cells. (A) Immunofluorescence staining of BV2 cells with IL-4 treatment. (B) Protein expression of Arg-1 in BV2 cells with IL-4 treatment via western blots. (C) Micrograph of obtained nanoparticles by TEM. Scale bar = 50 nm. (D) Diameter of BV2 cells derived extracellular vesicles measured by NTA. (E) Markers of extracellular vesicles, including Alix, TSG101, and CD63, were evaluated by western blot.


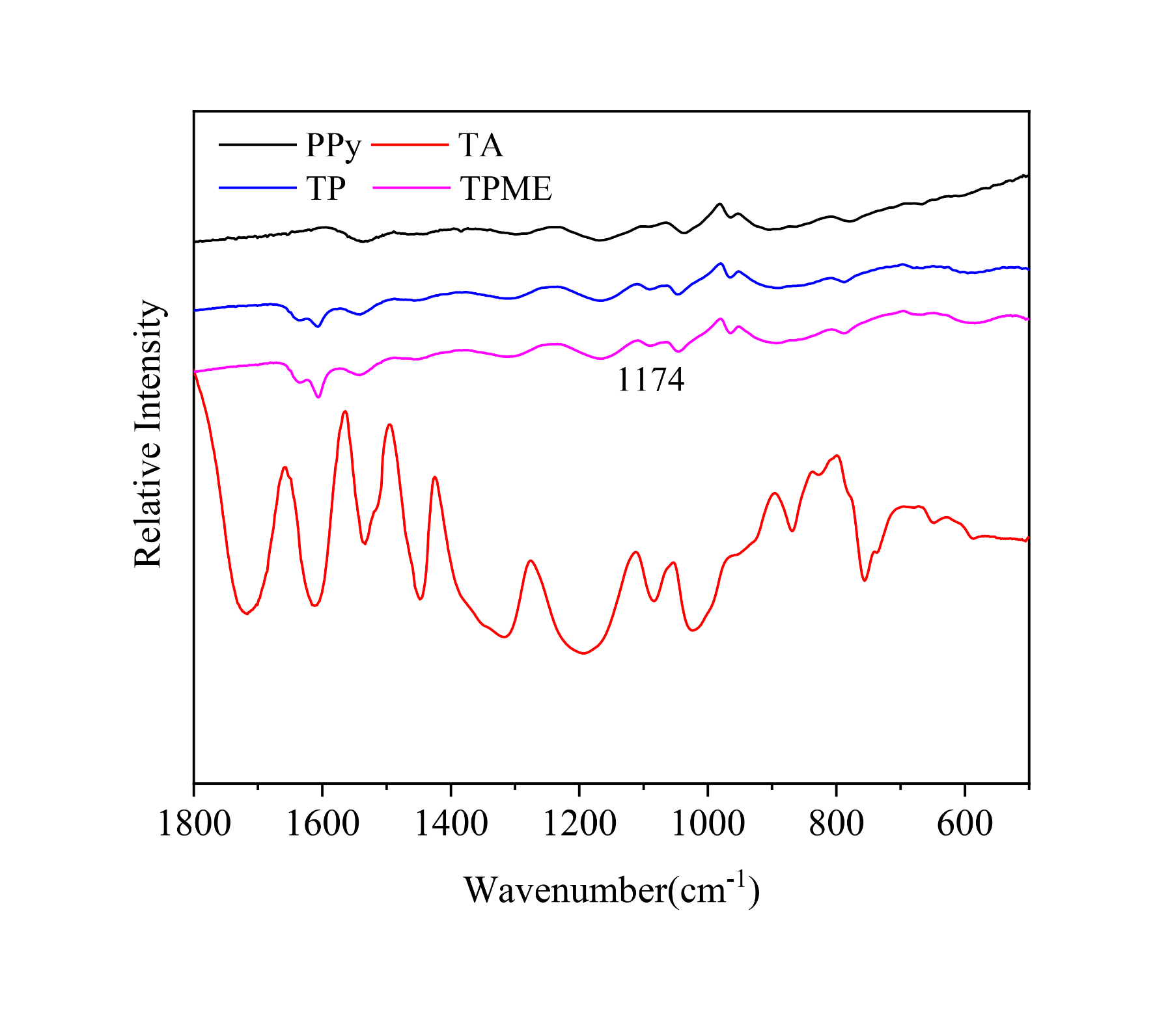


Fig. S2 FT-IR spectra of PPy, TA, TP, and TPME.


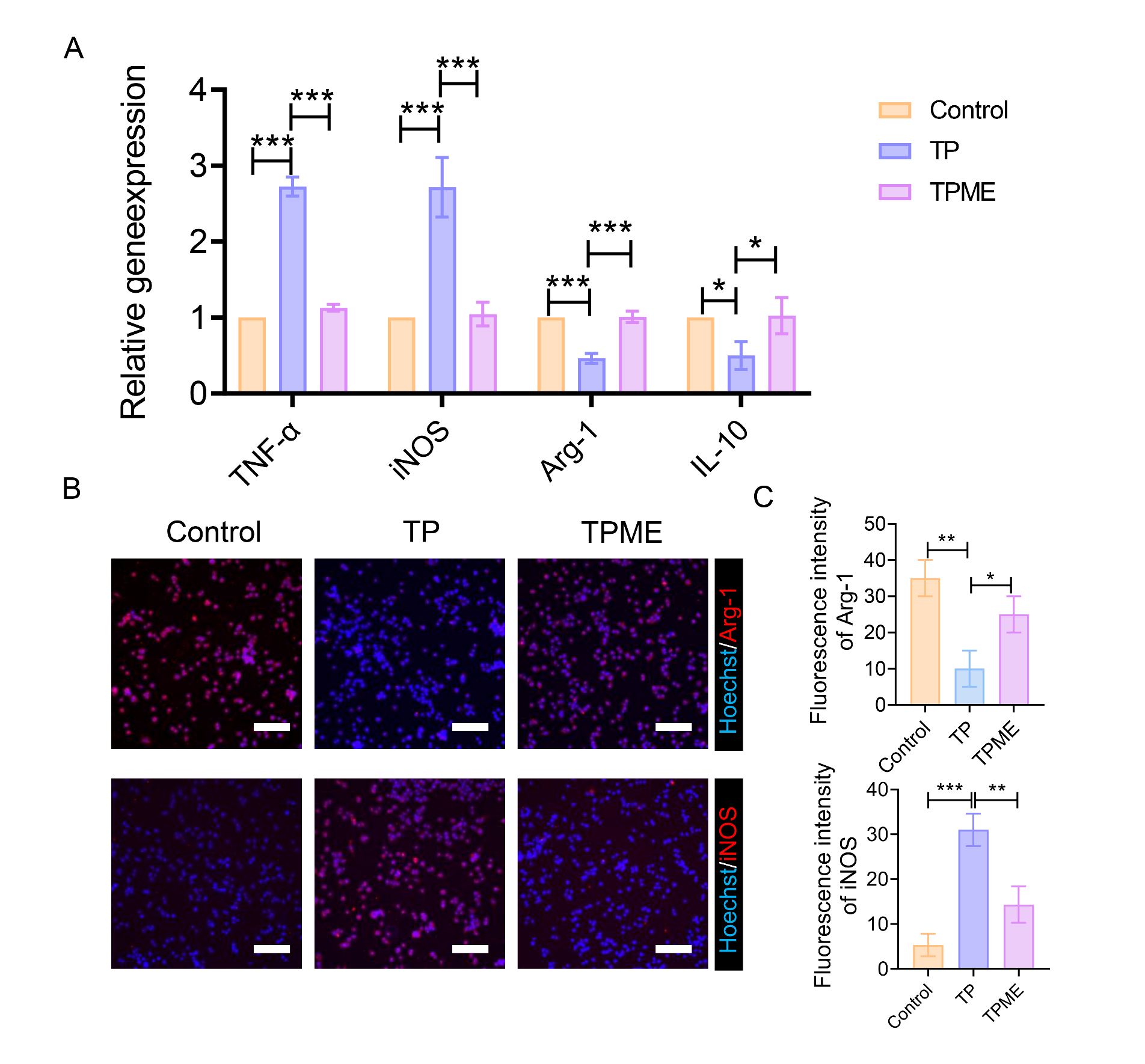


Fig. S3 TPME stimulated the polarization of BV2 cells from M1 to M2 phenotype. (A) Gene expression profiles of BV2 cells cultured on different samples for 3 days (n = 3). (B) Immunofluorescence (IF) images of Arg-1-positive and iNOS-positive BV2 cells cultured on each group. Scale bar = 100 μm. (C) Quantitative analysis of the fluorescence intensity of Arg-1 and iNOS (n = 3). Statistical analysis was performed using ANOVA followed by Tukey's test (*p < 0.05, **p < 0.01, and ***p < 0.001).

| Table S1. Primer sequences used for each gene are shown below. | | |  |
| --- | --- | --- | --- |
| Target | Forward | Reverse |  |
| NF | GTTCCGAGTGAGGTTGGACC | CCGCCGGTACTCAGTTATCTC |  |
| GAP43 | ATAACTCCCCGTCCTCCAAGG | GTTTGGCTTCGTCTACAGCGT |  |
| Arg-1 | CTCCAAGCCAAAGTCCTTAGAG | GGAGCTGTCATTAGGGACATCA |  |
| IL-10 | CTTACTGACTGGCATGAGGATCA | GCAGCTCTAGGAGCATGTGG |  |
| iNOS | GTTCTCAGCCCAACAATACAAGA | GTGGACGGGTCGATGTCAC |  |
| TNF-α | CGAGTGACAAGCCTGTAGCC | ACAAGGTACAACCCATCGGC |  |
| GAPDH | AGCCCAGAACATCATCCCTG | CACCACCTTCTTGATGTCATC |  |

| Product  Name | Species  Reactivity | Dilution  (cell/tissue) | Molecular  weight | Source |
| --- | --- | --- | --- | --- |
| GAPDH | Rabbit | 1:1000 | 37 kDa | CST, America |
| Tuj-1 | Mouse | 1:1000/1:200 | 55 kDa | Abcam, England |
| GFAP | Rabbit | 1:1000/1:200 | 50 kDa | Abcam, England |
| NF | Rabbit | 1:1000/1:200 | 102 kDa | CST, America |
| NeuN | Rabbit | 1:200 | 50 kDa | Abcam, England |
| MBP | Mouse | 1:1000/1:200 | 20 kDa | Abcam, England |
| GAP43 | Rabbit | 1:1000 | 48 kDa | Genetex, America |
| Arg-1 | Rabbit | 1:1000 | 35 kDa | GeneTex, America |
| iNOS | Rabbit | 1:1000 | 131 kDa | GeneTex, America |
| PTEN | Rabbit | 1:1000 | 54 kDa | CST, America |
| PI3K | Rabbit | 1:1000 | 85 kDa | CST, America |
| p-PI3K | Rabbit | 1:1000 | 85 kDa | CST, America |
| AKT | Rabbit | 1:1000 | 60 kDa | CST, America |
| p-AKT | Rabbit | 1:1000 | 60 kDa | CST, America |
| mTOR | Rabbit | 1:1000 | 289 kDa | CST, America |
| p-mTOR | Rabbit | 1:1000 | 289 kDa | CST, America |
| P70S6K | Rabbit | 1:1000 | 70 kDa | CST, America |
| p-P70S6K | Rabbit | 1:1000 | 70 kDa | CST, America |
| CD63 | Rabbit | 1:1000 | 26 kDa | Abcam, England |
| TSG101 | Rabbit | 1:1000 | 46 kDa | Abcam, England |
| Alix | Rabbit | 1:1000 | 96 kDa | Proteintech, America |

Table S2. Information about primary antibodies used.
